# Editorial: Rising stars in neuro-oncology and neurosurgical oncology 2021

**DOI:** 10.3389/fonc.2022.940063

**Published:** 2022-08-16

**Authors:** Lincoln Edwards, Ahmed Habib, Pascal O. Zinn

**Affiliations:** ^1^ Department of Neurosurgery, University of Pittsburgh Medical Center, Pittsburgh, PA, United States; ^2^ Hillman Cancer Center, University of Pittsburgh Medical Center, Pittsburgh, PA, United States

**Keywords:** neuro-oncology, neurosurgical oncology, oncology, rising, stars

Brain tumors remain at the top of the deadliest and most debilitating cancers that afflict humans to date. The gold standard of care for such tumors has been static over the years and consequently, the prognosis remains grim. Accordingly, the need for advancement in the management paradigm is of the utmost importance. Investigators and scientists all over the world are working tirelessly to unlock the mysteries of the molecular biology of these tumors. Adjunct surgical modalities are being developed and refined with the aim of helping clinicians achieve better clinical outcomes that will ultimately result in better survival benefits for brain cancer patients. In this editorial, we will summarize the seminal efforts of the authors who contributed to this important Research Topic.

This Research Topic focuses on the management paradigm of neurooncology patients. Namely, advances in intraoperative care, perioperative care, as well as advances in *in-vitro* brain tumor modeling which represent the most up to date basic and translational research with the goal of improving treatment and patient outcome for brain cancer patients.

## Intraoperative patient care

Giammalva et al. described in detail the benefits of the current applications of intraoperative ultrasound in brain tumor surgery. They have shown that intraoperative ultrasound represents a feasible and reliable non-invasive surgical modality that allows the surgeon to obtain real-time visual feedback of the spatial configuration of neuroanatomy and extent of resection of gliomas. The authors also pointed out the invaluable benefit of intraoperative ultrasound which takes into consideration the possibility of brain shift that occurs routinely during brain surgery. Furthermore, recent studies have emerged that suggest that the integration between conventional intraoperative ultrasound and the neuronavigational system is possible and yielded similar real-time visual feedback that accounts for brain shift during tumor resection. Interestingly, this same group described the role of a combination of radiofrequency ablation, vertebral reinforcement, and transpedicular fixation in the management of spinal metastatic tumors (Giammalva et al.). In their 54 patient series with thoracolumbar vertebral body metastases the mean postoperative visual analog scale (VAS) score was 7.81/10 representing a significant subjective pain reduction. Furthermore, the VAS score decreased over 6 months postoperatively (to 2.50/10). The data from this series suggested that the combined treatment model resulted in a significant pain reduction (p < 0.05); moreover, their proposed combination therapy represents a safe and effective treatment modality for the management of spinal metastases that resulted in a long-term beneficial clinical outcome in terms of pain relief.

## Perioperative patient care

In the study by Rosenstock et al. an MRI-based risk assessment was proposed for patients with incompletely resected subcortical brain metastases. The authors demonstrated that deep subcortical brain metastases (≥5mm) exhibiting diffuse contrast enhancement were associated with the highest incidence of unintended subtotal resection. The proposed MRI-based assessment allows for a personalized risk calculation for each patient and as a result, allows for a patient specific approach to resection subsequently resulting in better overall survival for these patients. Furthermore, Yan et al. explored the role of hypofractionated stereotactic radiotherapy in radiotherapy naïve patients series with small to moderate-sized brain metastases (60 patients with 133 metastatic lesions). The cohort median survival was 20.5 months and the rate of local failure at 12 months of follow-up was 17.8% which is highly comparable to the rates of whole-brain conventional radiation therapy. In general, hypofractionated radiotherapy is a relatively newer approach that has been adopted for a wide range of brain and skull base tumors that allows for a lesser logistic burden to healthcare facilities as well as offering a radiotherapy treatment paradigm with lesser side effects than conventional high-dose radiotherapy. Other studies are available that suggest the beneficial outcome of this newly adopted radiotherapy technique. Nevertheless, further multicentric studies investigating the endpoints of such a technique are warranted.

## *In-vitro* brain tumor modeling

The Linkous group -which represents an expert group in modeling brain tumors using cerebral organoids- described the feasibility of using cerebral organoids as an experimental invasion platform to model small cell lung cancer (SCLC) brain metastasis (Quaranta and Linkous). The authors have adopted a novel and revolutionary approach in their model which they referred to as the “systems approach” by which they were able to produce mathematical and computational models based on the *ex vivo* organoid cultures and in turn apply these models to clinical management guidelines. The benefit of this model is that it represents the first human model of SCLC. Mouse models have provided some benefits in deciphering SCLC subtypes; however, this has yet to be recapitulated in a human model. The SCLC model presented here provides the gateway to explore genetic manipulations, SCLC subtype characterization, and drug therapy. No doubt that the fields of *in-vitro* brain modeling have gained most of the limelight in the field of neuroscience and cancer biology during the last decade. Moreover, investigators are using these models to mirror a wide spectrum of brain conditions and tumors (glioblastoma, low-grade gliomas, brain metastasis, etc).

Advances in the field of neurooncology are becoming more central to improving patient care. The emerging efforts aiming to innovate and refine novel clinical and research approaches are showing results. Be that as it may, pushing the envelope is still slow and the need to further develop and refine is highly encouraged and warranted. The ultimate goal of these seminal efforts is to render brain and spinal cord primary and secondary tumors as highly manageable diseases and achieve the maximum survival outcomes possible for them.

## Author contributions

All authors listed have made a substantial, direct, and intellectual contribution to the work, and approved it for publication.

## Acknowledgments

We thank the authors of the articles published as a part of this Research Topic for their valuable contributions.

## Conflict of interest

The authors declare that the research was conducted in the absence of any commercial or financial relationships that could be construed as a potential conflict of interest.

## Publisher’s note

All claims expressed in this article are solely those of the authors and do not necessarily represent those of their affiliated organizations, or those of the publisher, the editors and the reviewers. Any product that may be evaluated in this article, or claim that may be made by its manufacturer, is not guaranteed or endorsed by the publisher.

